# Clinical and radiographic evaluation of Ponseti method for neonate congenital clubfoot

**DOI:** 10.3389/fped.2025.1619908

**Published:** 2025-06-11

**Authors:** Hai Jiang, Tao Li

**Affiliations:** Pediatric Orthopedics Department, Northwest Women’s and Children’s Hospital, Xian, China

**Keywords:** congenital clubfoot, Ponseti method, radiographic outcomes, talar length, talar height, calcaneus inclination angle, Meary's angle, talocalcaneal angle

## Abstract

**Objective:**

The objective of this study was to evaluate the clinical and radiographic outcomes of children with congenital clubfoot following treatment with the Ponseti method.

**Methods:**

We conducted a retrospective analysis of radiographic data from 20 children (12 males and 8 females) aged between 2 years 6 months and 7 years who underwent Ponseti method treatment for congenital clubfoot. The beginning treatment age was below 4 weeks after birth. The study included bilateral ten cases and unilateral ten cases. There were 20 cases which age matched normal feet in control group. The Pirani scoring system was used to assess the severity of the clubfeet. The average Pirani score was 5.5 (4.5–6). Radiographic measurements were taken from post-treatment images and compared to normative values. Talar length, talar height, calcaneus inclination angle, Meary's angle, and talocalcaneal angle were measured. Paired *t*-tests and effect size (*r*) were used to evaluate the effectiveness of the treatment.

**Results:**

All the patients were followed up at average 44 months (30–84 months). Functional, plantigrade feet with adequate mobility were achieved in all patients. The average Pirani score was 0.075 (0–0.5) at the end of the follow- up. Bilateral clubfoot exhibited significantly shorter talar length, lower talar height, smaller talocalcaneal angles, smaller calcaneus inclination angles and smaller Meary's angles compared to normal feet. Unilateral clubfeet exhibited a significantly greater talar height and a slightly higher calcaneus inclination angles compared to bilateral clubfeet. There were no significant differences observed in talar length, talocalcaneal angle, or Meary's angle between bilateral and unilateral clubfoot groups.

**Conclusions:**

The Ponseti method has been proven to be highly effective in achieving functional correction in children with congenital clubfoot. However, radiographic images in this study revealed differences in tarsal bone morphology. Therefore, further long-term studies are necessary to evaluate the durability of these corrections and their impact on functional mobility in adulthood. The future study should also aim to clarify the causes of observed morphological alterations.

## Introduction

Congenital clubfoot is one of the most common musculoskeletal birth defects, affecting approximately 1 in 1,000 live births worldwide, with regional variations in prevalence (1). This deformity is characterized by a complex foot malpositions: forefoot adduction, varus, middle foot cavus and hind foot equinus. Pathologically, it involves structural abnormalities of the talus and calcaneus, along with soft tissue contractures (2). If left untreated, congenital clubfoot results in significant functional impairment, including gait abnormalities and lifelong disability.

**Figure 1:**
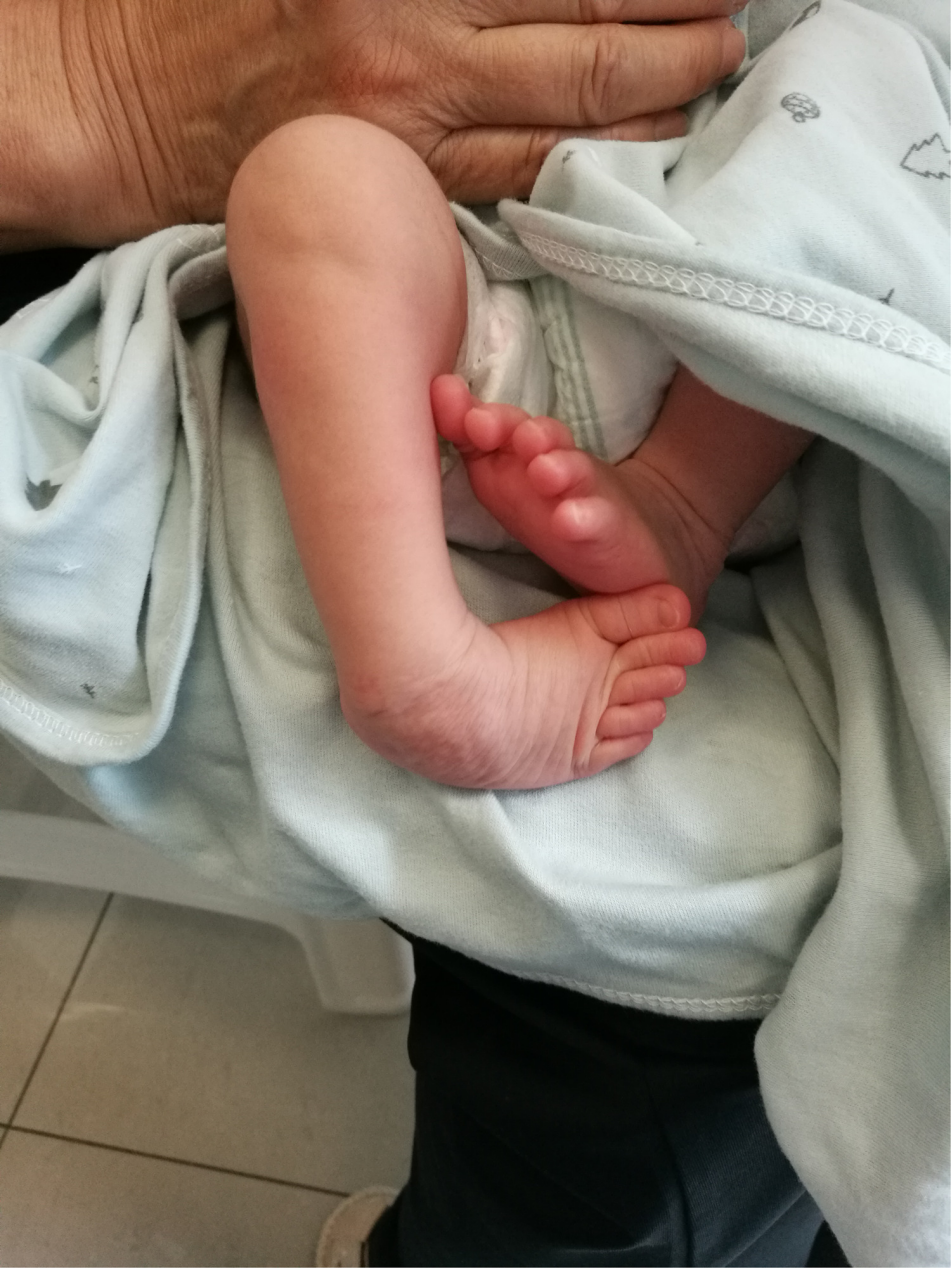
The right foot exhibited the classic deformity of clubfoot.

**Figure 2:**
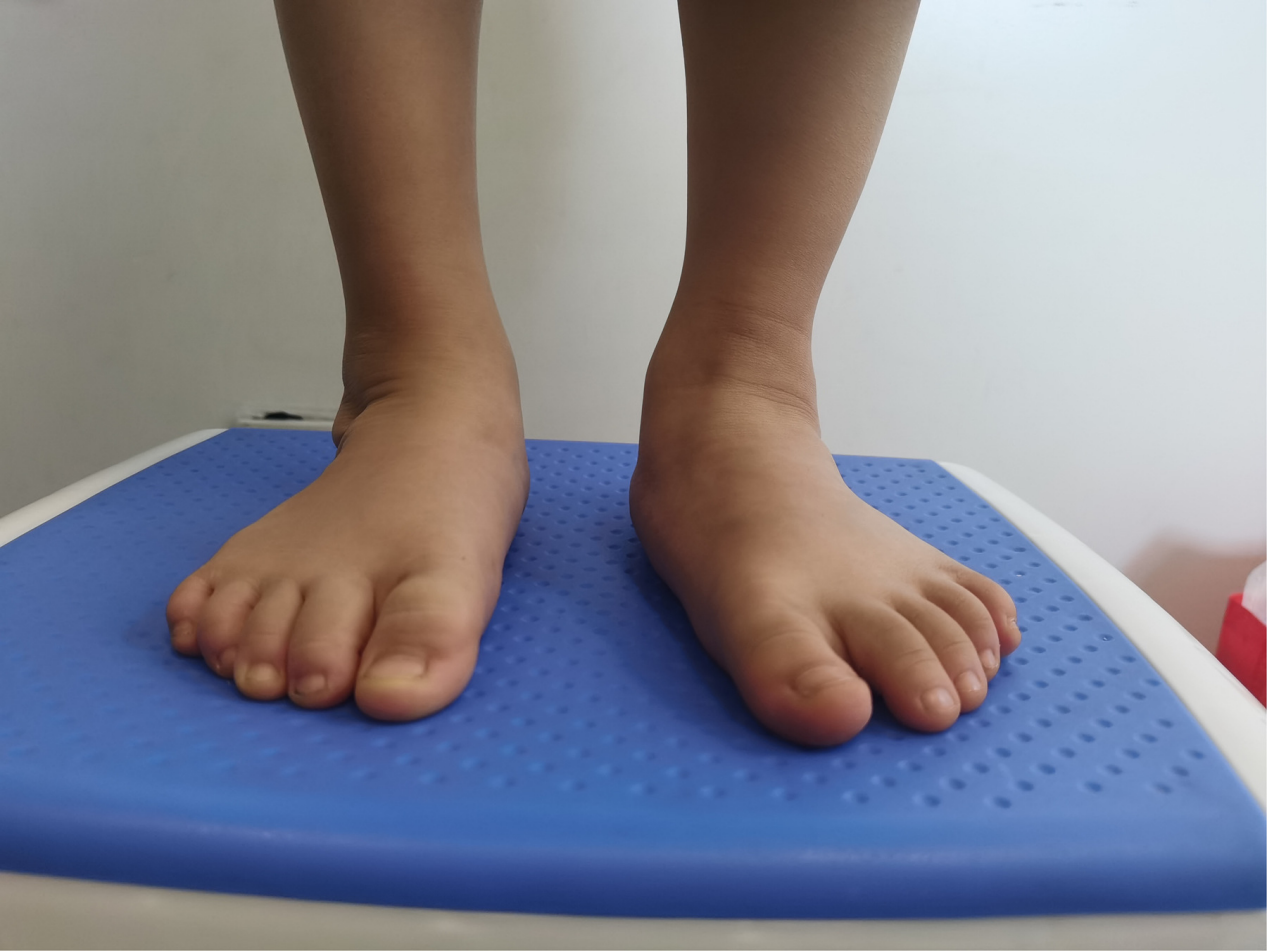
After 5 years follow-up, the foot had a normal appearance.

**Figure 3:**
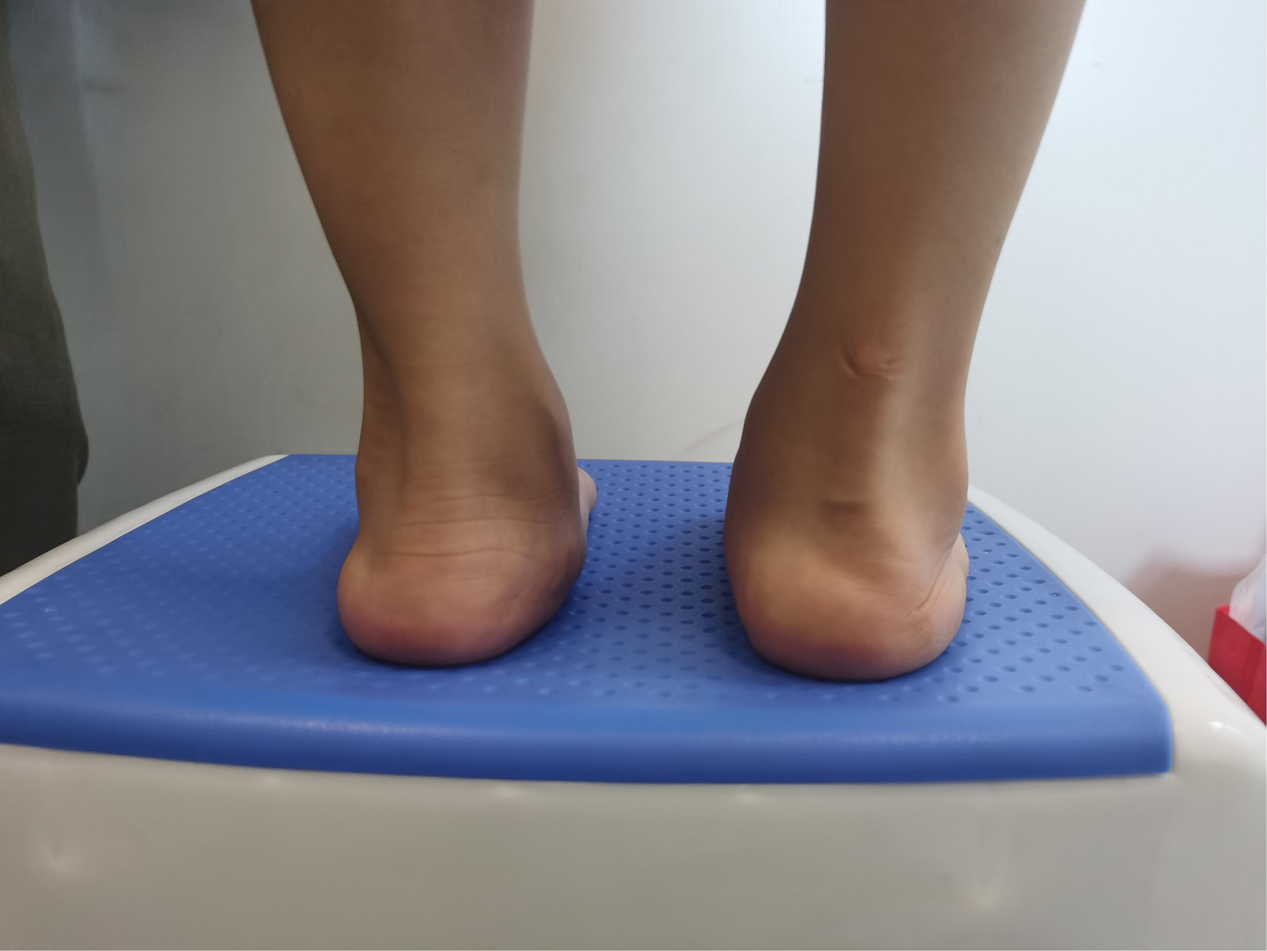
After five years follow-up, the foot had a normal appearance.

**Figure 4:**
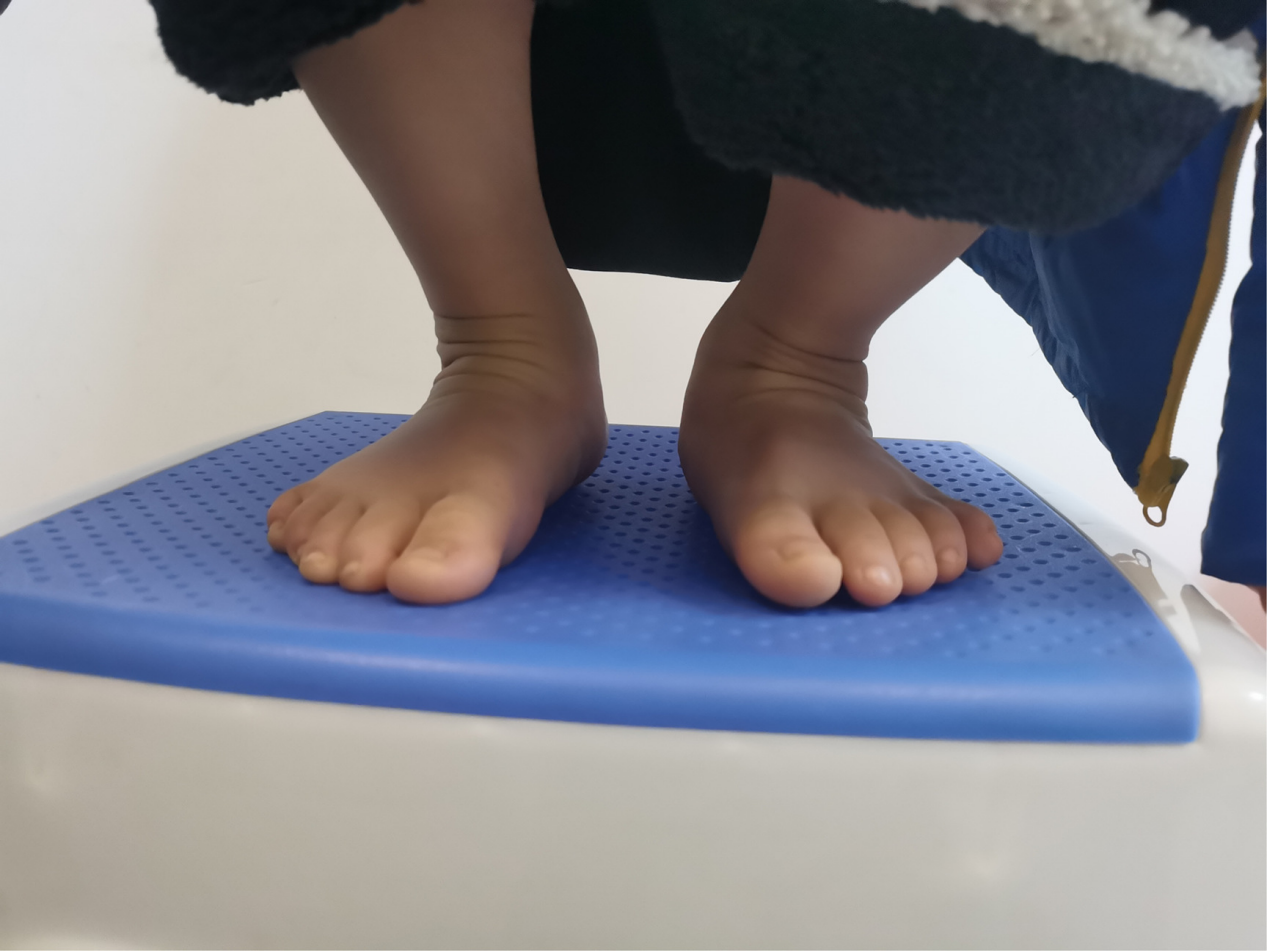
The ankle's dorsiflexion function was normal.

**Figure 5:**
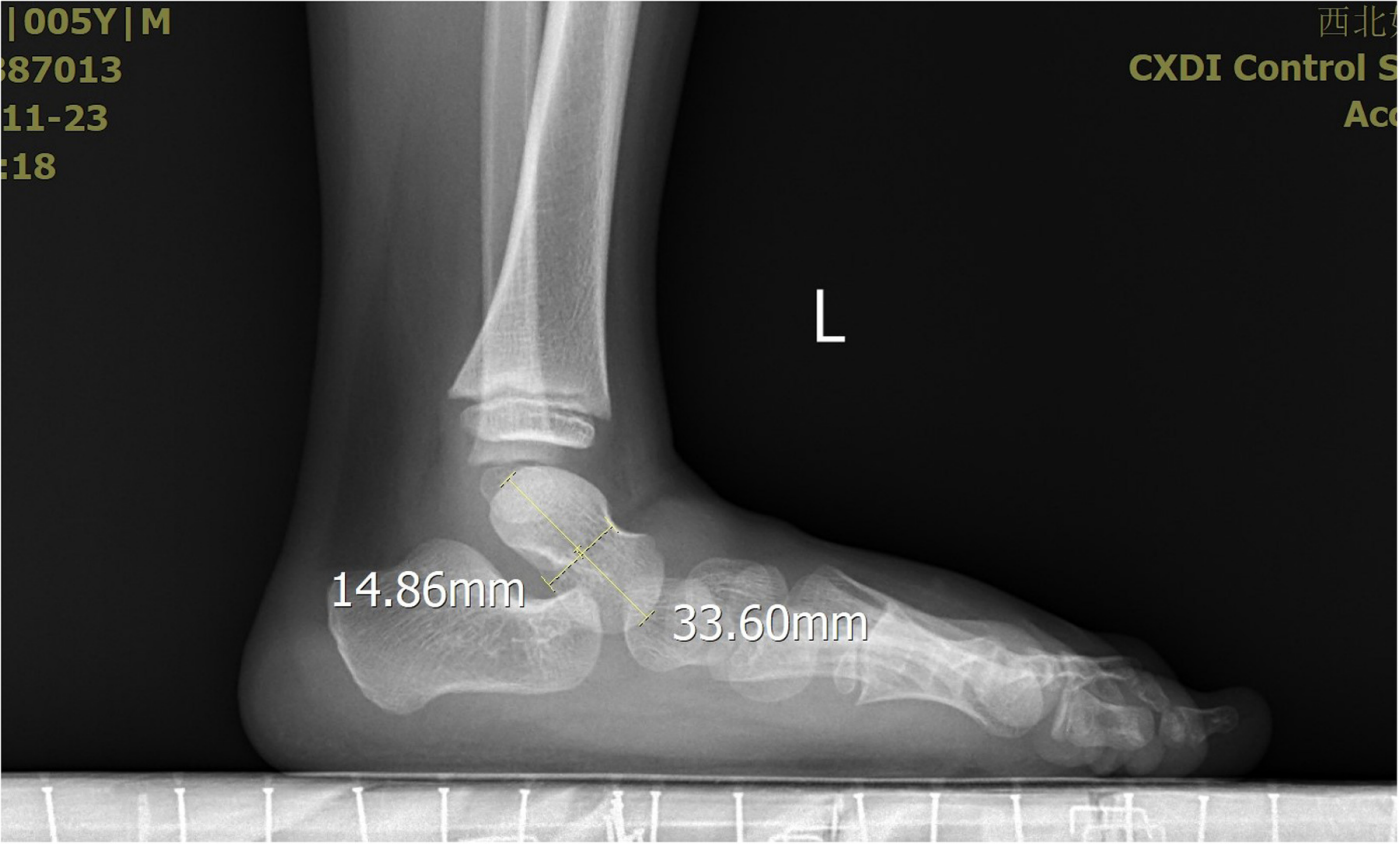
The X-ray demonstrated the measurement of the talus length and height.

**Figure 6:**
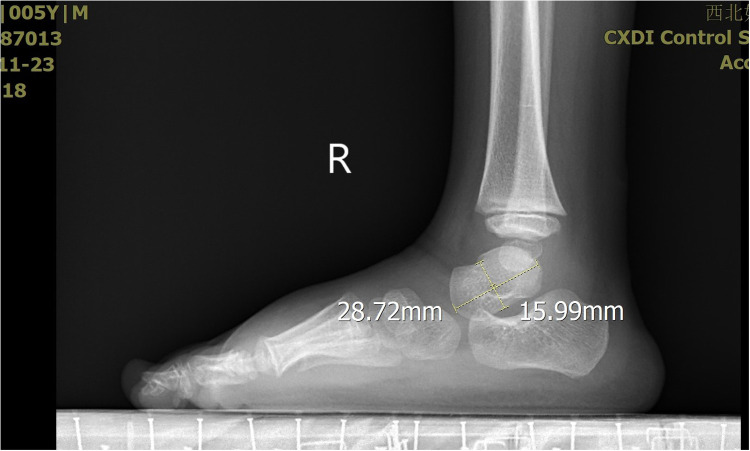
The X-ray demonstrated the measurement of the talus length and height.

**Figure 7:**
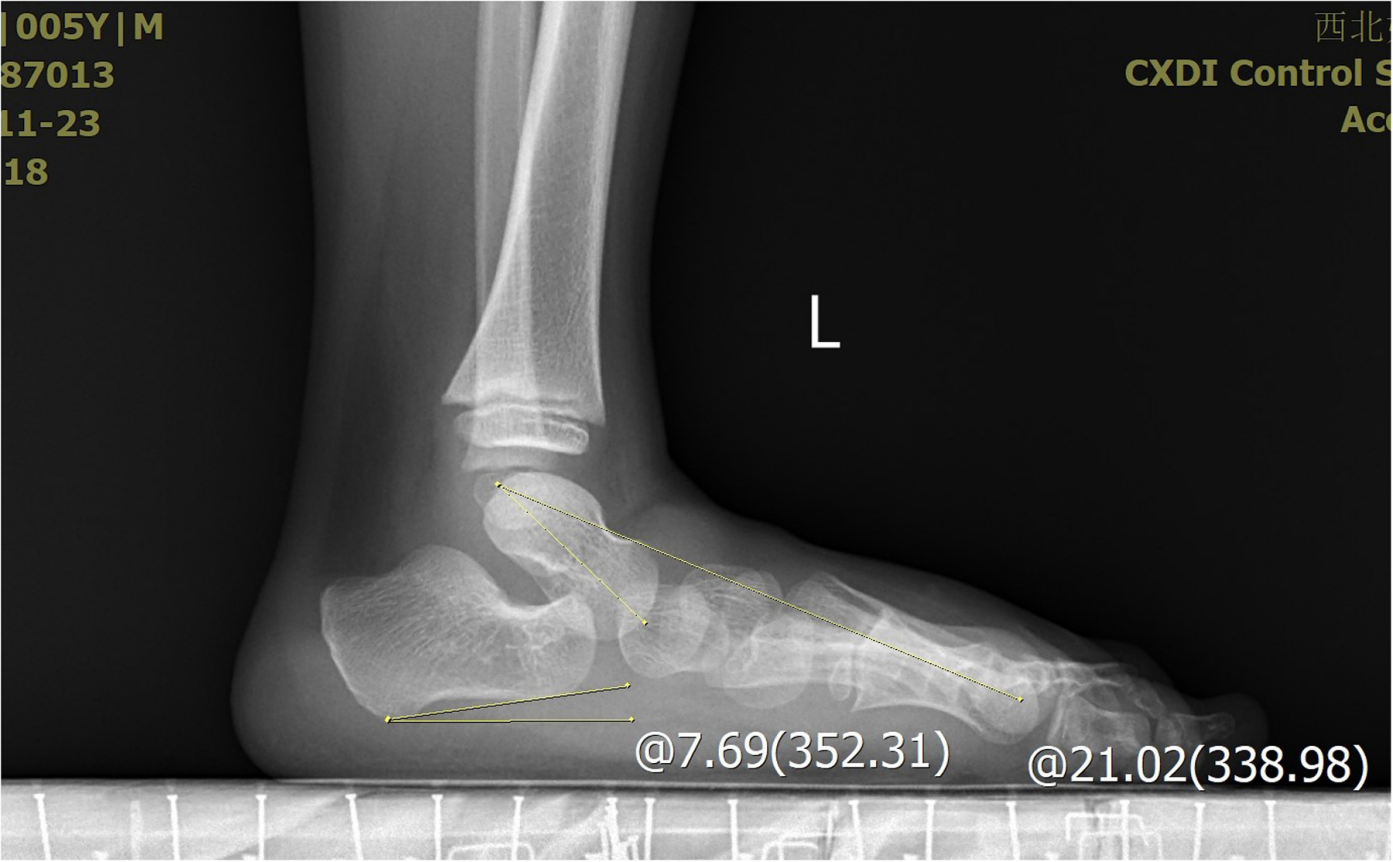
The X-ray demonstrated the measurement of the calcaneus inclination angle and Meary's angle.

**Figure 8:**
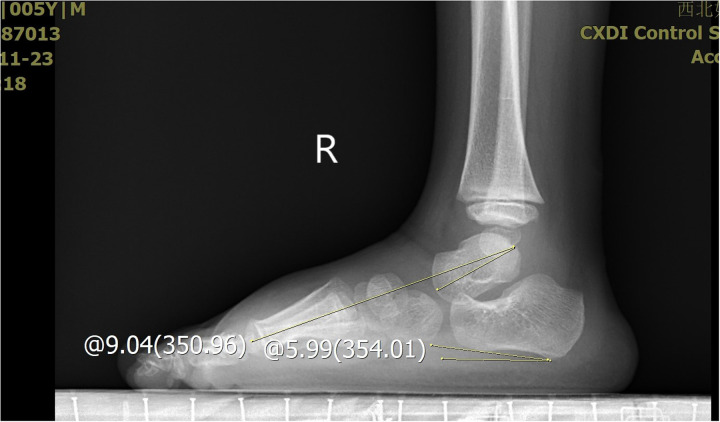
The X-ray demonstrated the measurement of the calcaneus inclination angle and Meary's angle.

**Figure 9:**
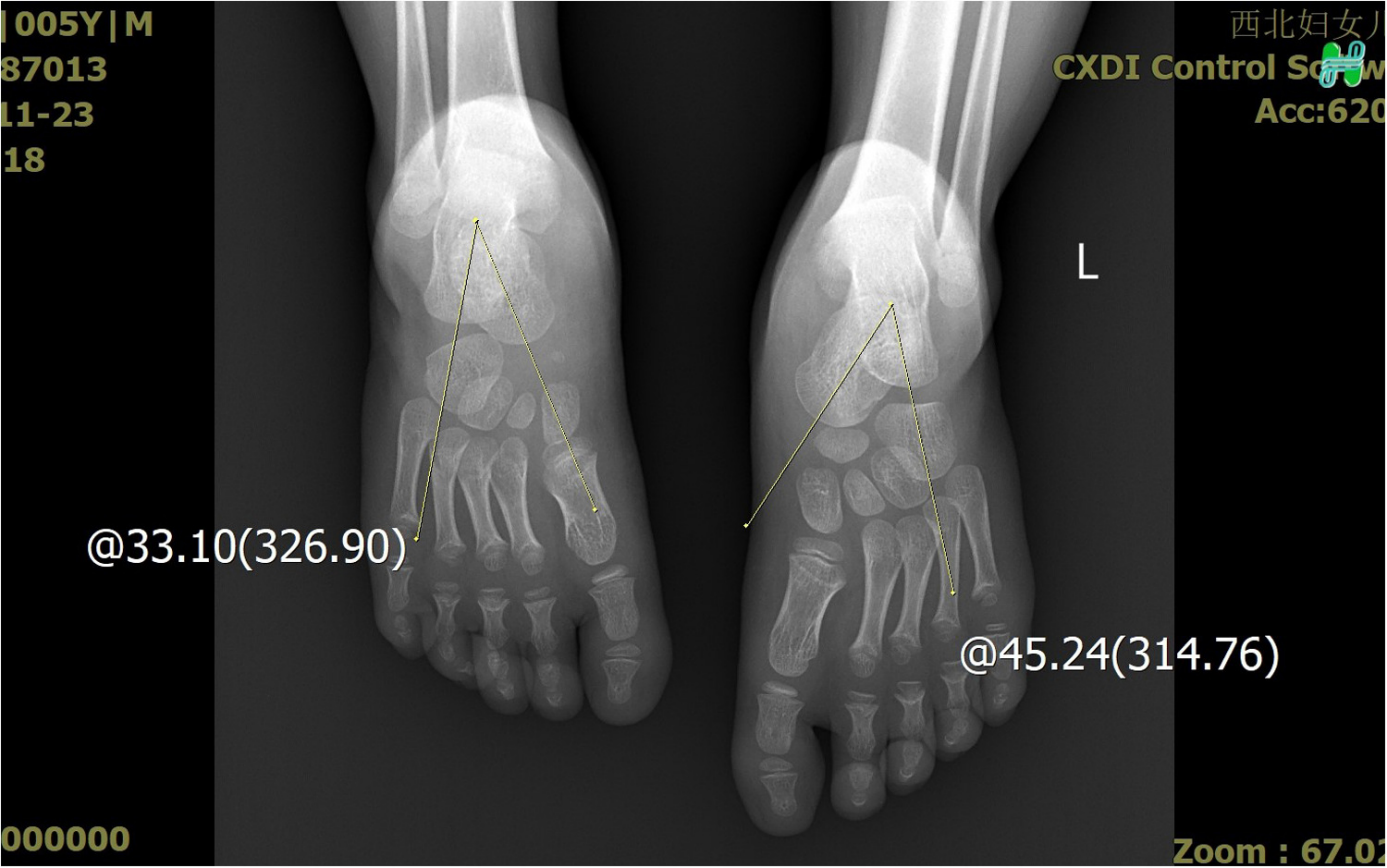
The X-ray demonstrated that the affected talocalcaneal angle was smaller than the normal side.

**Figure 10:**
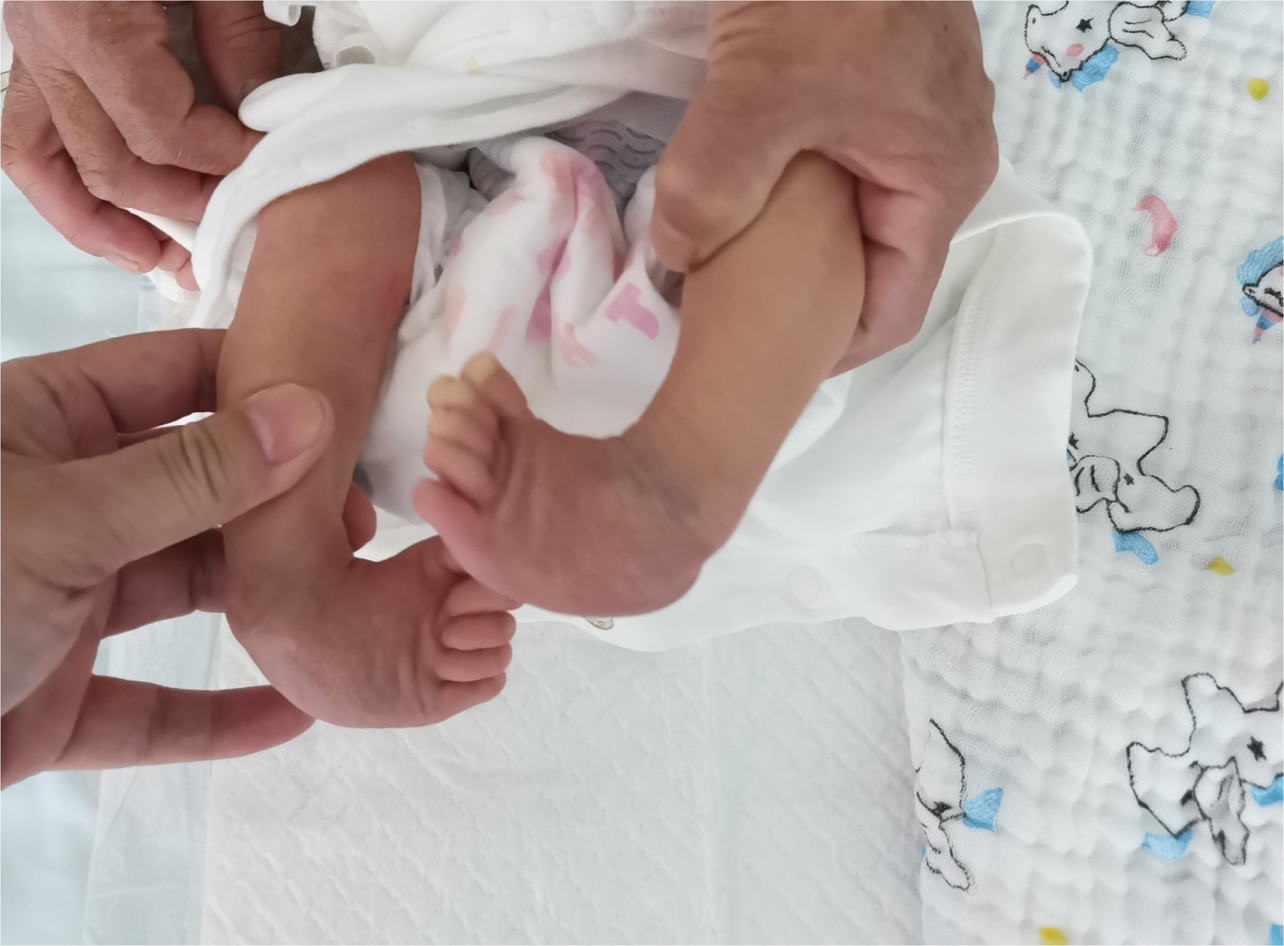
The both feet exhibited the classic deformity of clubfoot.

**Figure 11:**
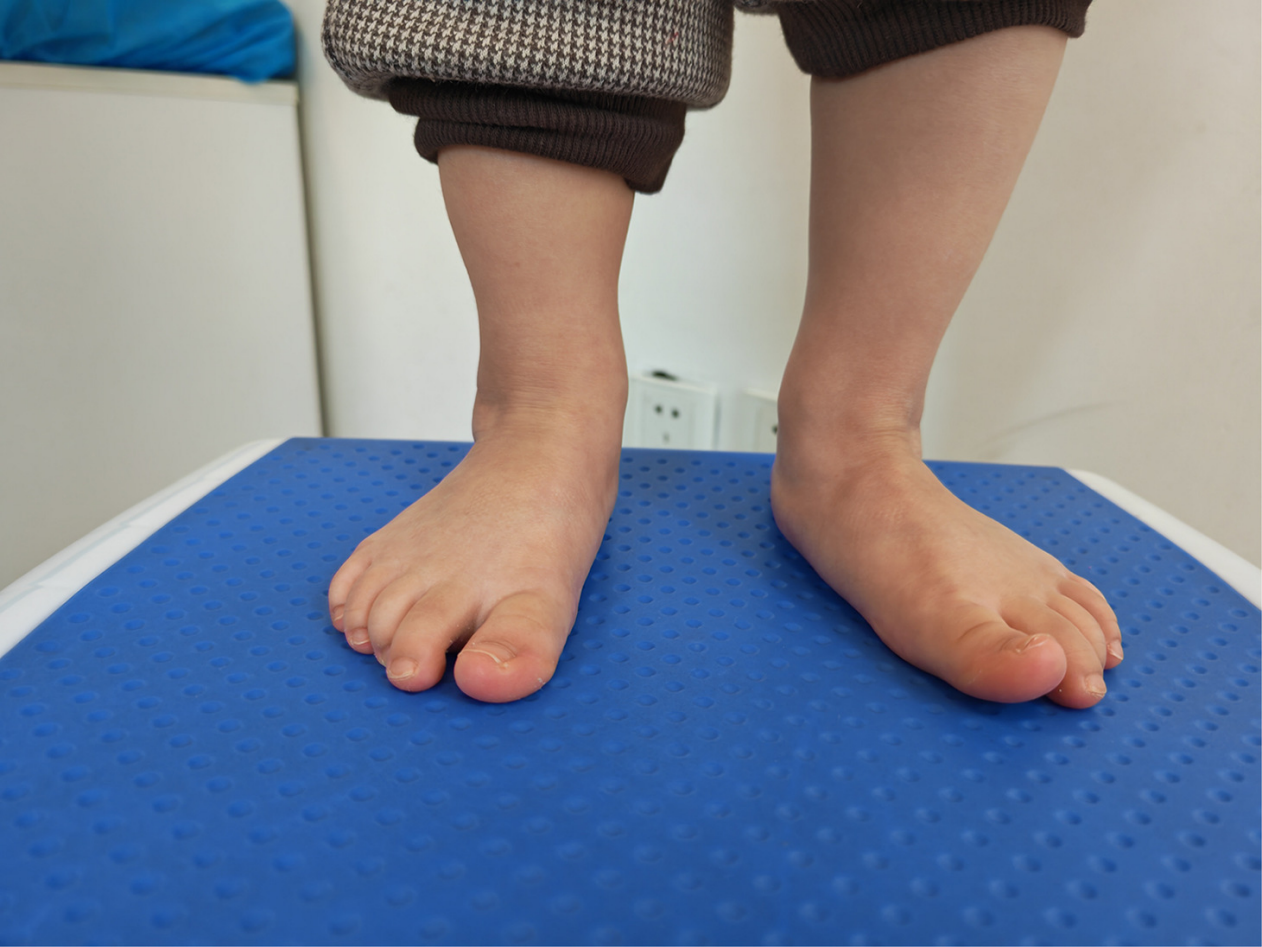
After three years follow-up, the feet had a normal appearance.

**Figure 12:**
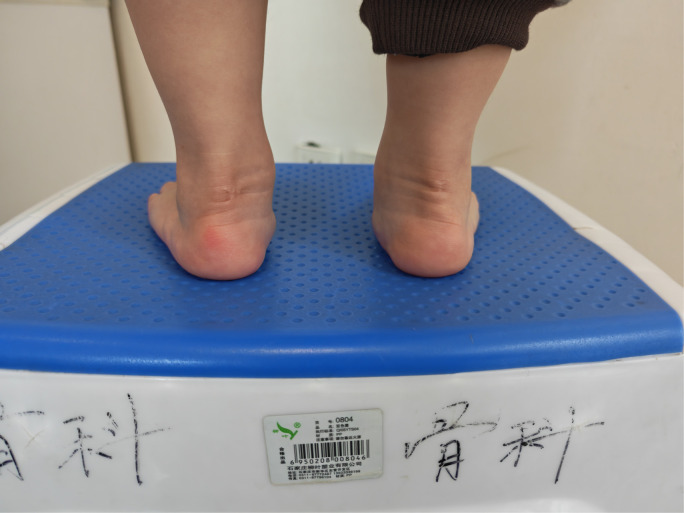
After three years follow-up, the feet had a normal appearance.

**Figure 13:**
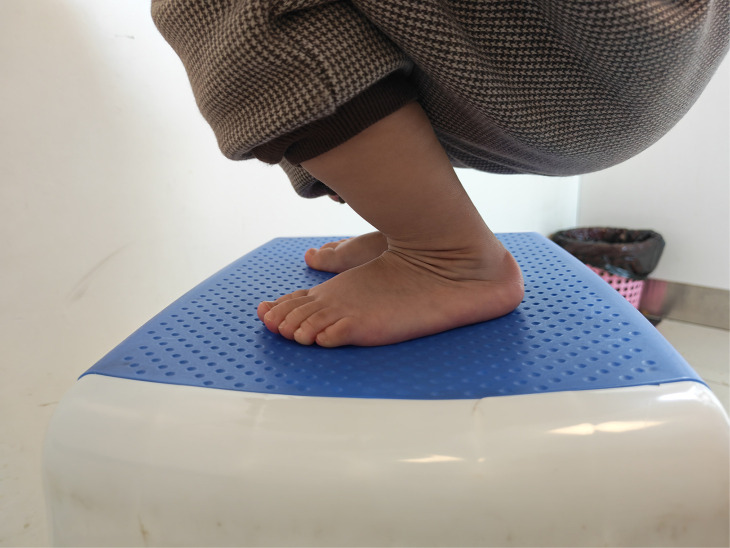
The both ankle's dorsiflexion function was normal.

**Figure 14:**
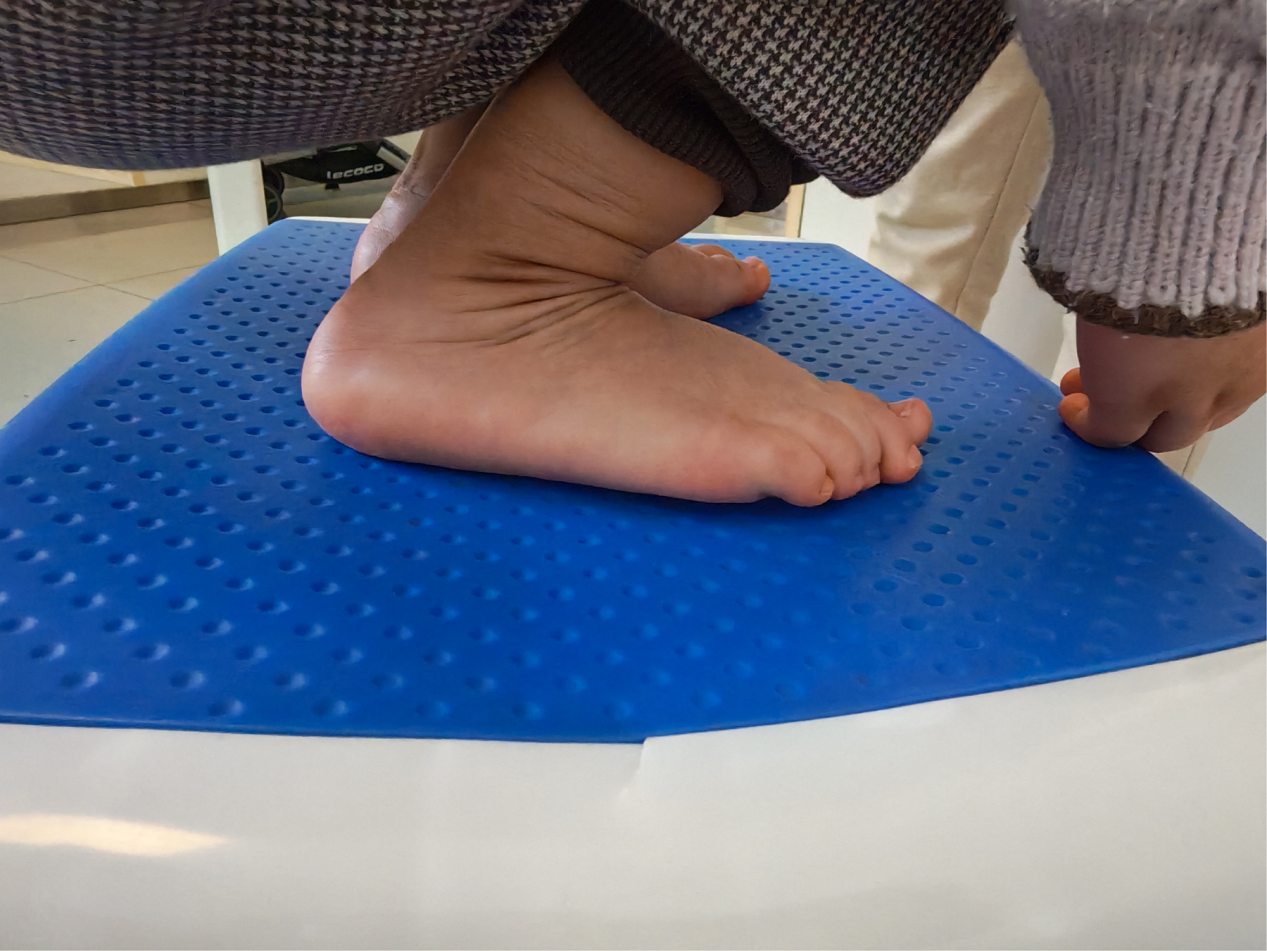
The both ankle's dorsiflexion function was normal.

**Table 1:** Radiological measurements in bilateral vs. unilateral clubfoot.

Radiological measurement	Bilateral clubfoot (*n* = 10)	Unilateral clubfoot (*n* = 10)	*p*-value	Effect size (*r*)
Talar length (mm)	30.5 ± 5.2	32.1 ± 4.8	0.08	0.25
Talar height (mm)	14.2 ± 2.1	15.3 ± 2.3	0.02	0.45
Talocalcaneal angle (°)	12.3 ± 4.5	14.2 ± 5.1	0.12	0.30
Calcaneus inclination angle (°)	7.8 ± 3.2	9.2 ± 3.5	0.05	0.40
Meary's angle (°)	33.5 ± 6.8	35.1 ± 7.2	0.18	0.20

**Table 2:** Radiological measurements in bilateral clubfoot vs. normal feet.

Radiological measurement	Bilateral clubfoot (*n* = 10)	Normal feet (*n* = 20)	*p*-value	Effect size (*r*)
Talar length (mm)	30.5 ± 5.2	35.0 ± 4.5	<0.01	0.50
Talar height (mm)	14.2 ± 2.1	17.5 ± 2.5	<0.001	0.60
Talocalcaneal angle (°)	12.3 ± 4.5	20.0 ± 3.2	<0.001	0.70
Calcaneus inclination angle (°)	7.8 ± 3.2	12.0 ± 2.8	<0.001	0.65
Meary's angle (°)	33.5 ± 6.8	38.0 ± 5.5	<0.01	0.45

Data are presented as mean ± standard deviation.

*p*-values are derived from the independent sample *t*-test or paired sample *t*-test.

Effect size (*r*) is calculated to quantify the magnitude of differences.

The Ponseti method, developed by Dr. Ignacio Ponseti in the 1950s, has revolutionized the management of clubfoot due to its non-invasive approach and high efficacy. This technique combines several steps including serial gentle manipulations, weekly casting, and a percutaneous Achilles tenotomy in most cases, followed by bracing to prevent relapse. Compared to traditional surgical interventions, the Ponseti method has a high success rate exceeding 90% with minimal complications, a low recurrence risk, and superior long-term functional outcomes (3). Its benefits lie in avoiding extensive soft tissue dissection, preserving native joint mobility, and enabling early functional recovery, making it the gold standard for primary treatment (4).

Despite its clinical satisfied outcomes, radiographic evaluation remains critical for objectively assessing anatomical correction. These imaging parameters are essential for verifying the normalization of bone relationships, detecting residual deformities, and guiding decisions on adjunct procedures (5). Furthermore, longitudinal imaging studies may help monitor relapse risks and validate the durability of corrections, ensuring optimal outcomes as children grow. Thus, this study integrates radiographic analysis to evaluate the structural efficacy of the Ponseti method in restoring foot anatomy and compare these data with normal control group.

## Methods

This retrospective study evaluated the efficacy of the Ponseti method in treating congenital clubfoot in 20 pediatric patients. Data were collected from medical records of patients treated in our hospital between June 2017 and June 2022. The study included 10 cases of bilateral clubfeet and 10 cases of unilateral clubfeet, with a mean age at the start of treatment of less than 4 weeks. Inclusion criteria were idiopathic CTEV diagnosed clinically. Exclusion criteria included non-idiopathic clubfeet, syndromic conditions, previous surgical intervention, or incomplete medical records.

The severity of clubfoot deformity was assessed using the Pirani scoring system. The mean score was 5.5 (4.5–6). Radiographic assessments were performed to evaluate the talar length, talar height, calcaneus inclination angle, Meary's angle and talocalcaneal angle after treatment at the end of the follow-up. These measurements were obtained from standardized x-ray images of the both feet.

All patients were treated using the Ponseti method, which consists of three main phases: manipulation and casting, Achilles tenotomy, and bracing. The affected feet were manually manipulated to correct the deformity, followed by application of long-leg plaster casts. The casts were changed weekly. The number of casts before tenotomy was 4 times in18 patients. There were 6 times in 2 patients because of the cast loose. The Achilles tenotomy procedure was conducted under general anesthesia, and a small incision was made to release the tight Achilles tendon. After achieving correction, patients were fitted with a foot abduction brace to maintain the corrected position. The brace was used full-time for the first 3 months, followed by nighttime use for at least 4 years to prevent recurrence.

## Statistical methods

Descriptive statistics were calculated for each radiological measurement in both bilateral and unilateral clubfoot groups. The mean ± standard deviation (SD) and median (interquartile range, IQR) were computed to summarize the data. This test is appropriate for comparing two independent groups with non-normally distributed data. The effect size (*r*) was calculated to quantify the magnitude of differences between groups.

Significance Level: A *p*-value of less than 0.05 was considered statistically significant.

## Results

All the patients were followed up for 30 m to 7 years, average time 44 months. The feet appearances and mobilities were observed and the feet x-ray pictures were taken (Figures 1–9). Functional, plantigrade feet with adequate mobility were achieved in all patients (Figures 10–14). Two patients (one bilateral boy and one unilateral girl) were not compliant with the brace and the equinus deformity relapse. The Achilles tenotomy procedure was conducted again. The average Pirani score was 0.075 (0–0.5) at the end of the follow- up. The talar length and height, calcaneus inclination angle, Meary's angle and talocalcaneal angle were observed.

## Statistical analysis results

Unilateral clubfoot exhibited a significantly greater talar height compared to bilateral clubfoot (*p* = 0.02; effect size *r* = 0.45). This suggests more pronounced hindfoot deformity in unilateral cases. Unilateral clubfoot had a slightly higher CIA, although the difference was only marginally significant (*p* = 0.05; effect size *r* = 0.40). No significant differences were observed in talar length, talocalcaneal angle, or Meary's angle between bilateral and unilateral clubfoot groups (*p* > 0.05) (Table 1).

Bilateral clubfoot exhibited significantly shorter talar length, lower talar height, smaller talocalcaneal angles, smaller CIAS and smaller Meary's angles compared to normal feet (Table 2).

## Discussion

The Ponseti method has been widely recognized as the gold standard for the treatment of congenital clubfoot due to its high efficacy and reproducibility. Numerous studies have demonstrated success rates exceeding 90% when the protocol is strictly followed, particularly when combined with appropriate bracing compliance (6). Long-term follow-up studies have corroborated its durability, with most patients achieving functional, pain-free feet into adolescence (7–9). The low recurrence rates observed in compliant populations further underscore its superiority over invasive surgical approaches, which are associated with higher risks of stiffness and degenerative joint changes (10).

Post-treatment morphological alterations of the talus, specifically reduced length and height, warrant careful consideration. These changes may stem from incomplete correction of the original deformity, residual medial displacement of the navicular, or altered mechanical loading patterns during the remodeling phase. In the present study, talar length was only minimally reduced to an average of 95% compared to the control group. On the other hand, talar height was highly reduced to 84% compared to normal feet (11). Our research results were familiar with the present study. The talar length was reduced to an average of 87% compared to the control group. The talar height was reduced to 81% compared to normal feet. The talus shape changed more flatten compared to normal feet. What cause the flat shape is not clear. After treatment of congenital talipes equinovarus in children using the Ponseti method, the appearance of a flattened talus may be due to several potential causes. One possibility is that excessive pressure applied during the casting process may lead to ischemic necrosis of the talus. The subsequent repair process could then result in a flattened shape, which was similar with the flat appearance of the femoral head following ischemic necrosis. Another potential cause is that prolonged immobilization with plaster may affect the normal development of the talus, leading to slow growth in both length and height, and consequently a flattened appearance. Further research is needed to determine the specific cause.

The Meary's angle is a valuable parameter for assessing mid-foot alignment. In our study, two patients exhibited a Meary's angle exceeding 5°, indicating residual high-arch deformity in the mid-foot, suggestive of undercorrection. The tension of the Achilles tendon can significantly influence the calcaneal inclination angle through its traction force. Specifically, two patients had a calcaneal inclination angle below 0°, which may reflect insufficient release of the Achilles tendon. However, despite this finding, both patients demonstrated satisfactory ankle dorsiflexion function.

Magnetic resonance imaging (MRI) has been extensively reported in the literature for assessing the morphology and alignment of the foot bones following Ponseti method treatment for clubfoot. MRI enables a more three-dimensional visualization of the tarsal bones, including the talus, calcaneus, and navicular bone, by providing multiplanar imaging in the coronal, sagittal, and axial planes. It also allows for a comprehensive evaluation of the positional relationships between the talus and the distal tibia, thereby offering a more detailed understanding of the relationships within the ankle joint and foot bones (12, 13). Guda et al. (14) reported the use of three-dimensional MRI to assess the morphological and alignment characteristics of the talus in clubfoot after treatment with the Ponseti method. Their study revealed that the volume of the talus on the affected side was significantly smaller than that on the contralateral side. Additionally, there were differences in the medial deviation angles of the talar head and neck between the affected and contralateral sides, and correlations were observed between talar deformity and the alignment of the navicular bone as well as the internal rotation angle of the distal tibiofibular joint. Ahmad et al. (15) measured various angles in the foot following accelerated Ponseti method treatment using MRI, confirming improvements in skeletal relationships. However, the use of magnetic resonance imaging (MRI) requires sedation and is associated with high costs, which limits its clinical application.

This study acknowledges several limitations. The single-center design with small sample size (*n* = 20) may limit generalizability, particularly regarding ethnic variations in pathoanatomy. The mean follow-up duration of 3 years 8 months remains insufficient to assess skeletal maturity-related changes or late-onset degenerative changes. Future investigations should prioritize multicenter collaborations to enhance statistical power and demographic diversity.

## Conclusions

The Ponseti method has been proven to be highly effective in achieving functional correction in children with congenital clubfoot. However, radiographic images in this study revealed differences in tarsal bone morphology. Therefore, further long-term studies are necessary to evaluate the durability of these corrections and their impact on functional mobility in adulthood. The future study should also aim to clarify the causes of observed morphological alterations.

## Data Availability

The raw data supporting the conclusions of this article will be made available by the authors, without undue reservation.
